# Systematic analysis and optimization of early warning signals for critical transitions using distribution data

**DOI:** 10.1016/j.isci.2023.107156

**Published:** 2023-06-16

**Authors:** Daniele Proverbio, Alexander Skupin, Jorge Gonçalves

**Affiliations:** 1 Luxembourg Centre for Systems Biomedicine, University of Luxembourg, 6 Avenue Du Swing, 4367 Belvaux, Luxembourg; 2 College of Engineering, Mathematics and Physical Sciences, University of Exeter, Exeter EX4 4QL, UK; 3 National Center for Microscopy and Imaging Research, University of California San Diego, Gilman Drive, La Jolla, CA 9500, USA; 4 Department of Physics and Material Science, University of Luxembourg, 162a Avenue de La Faiencerie, 1511 Luxembourg, Luxembourg; 5 Department of Plant Sciences, University of Cambridge, Cambridge CB2 3EA, UK

**Keywords:** Bioinformatics, Mathematical biosciences

## Abstract

Abrupt shifts between alternative regimes occur in complex systems, from cell regulation to brain functions to ecosystems. Several model-free early warning signals (EWS) have been proposed to detect impending transitions, but failure or poor performance in some systems have called for better investigation of their generic applicability. Notably, there are still ongoing debates whether such signals can be successfully extracted from data in particular from biological experiments. In this work, we systematically investigate properties and performance of dynamical EWS in different deteriorating conditions, and we propose an optimized combination to trigger warnings as early as possible, eventually verified on experimental data from microbiological populations. Our results explain discrepancies observed in the literature between warning signs extracted from simulated models and from real data, provide guidance for EWS selection based on desired systems and suggest an optimized composite indicator to alert for impending critical transitions using distribution data.

## Highlights

• Early warning signals (EWS) for critical transitions are still poorly performant
• Our framework assesses the performance of EWS in noisy deteriorating conditions
• Composite indicators are optimized to provide EWS using distribution data
• The results are tested on a wide class of models and empirical data

## Graphical abstract

**Figure:**
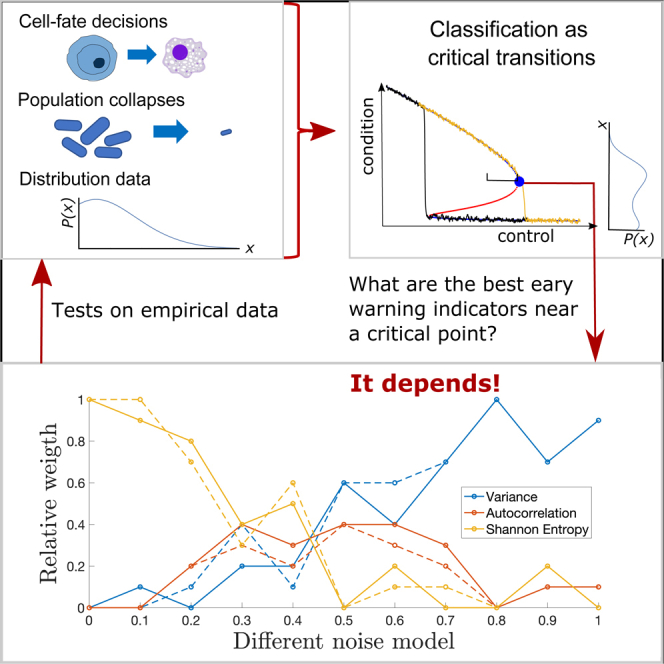

## Introduction

The dynamics of many complex systems is characterized by critical thresholds (tipping points) and abrupt shifts between alternative regimes.^1^^,^^2^ Various examples have been observed in diverse research fields and include collapses of ecosystems,^3^^,^^4^ sudden climate shifts^5^^,^^6^ or financial crashes.^7^^,^^8^ Abrupt regime shifts have particularly been theorized and observed in systems biology and medicine,^9^^,^^10^^,^^11^ at the onset of certain disease states like atrial fibrillation^12^ or epileptic seizures,^13^ as well as in biological processes like regulation of gene networks^14^^,^^15^ and cell fate decisions,^16^^,^^17^ including epithelial-mesenchymal transitions.^18^ Correctly detecting and alerting for these critical changes allows us to better understand complex developments and to anticipate dangerous outcomes. However, many such complex systems have not been fully characterized with mechanistic models, thus requiring simpler and more generic approaches to support data-driven estimates.

The critical transitions (CT) framework have been proposed to address tipping points using low-dimensional systems descriptions^19^ and associated early warning signals (EWS), computed from statistical indicators extracted from data like increasing variance, autocorrelation or coefficient of variation.^20^^,^^21^ These signs and derived indexes,^22^^,^^23^^,^^24^ in principle generic for broad classes of systems, have been tested and applied on biological, epidemiological and medical data with alternate success.^25^^,^^26^^,^^27^^,^^28^ Therefore, recent studies have recommended caution when attempting predictions based on EWS.^29^^,^^30^^,^^31^ Since there is an increasing interest for EWS in systems biology and biomedicine, it is thus compelling to provide a unified framework for the analysis and interpretation of such indicators, to determine in which cases they can be safely applied and to understand their limitations, in particular when considering the type of data that are usually available from systems biology experiments. In addition, going beyond univariate indicators will improve their performance in detecting and alerting for impending critical transitions.

In this work, we provide a systematic analysis of the CT framework and its associated EWS, to define their range of applicability and understand why discrepancies have been observed between theoretical predictions and experimental data.^32^^,^^33^ Systems biology is characterized by two main paradigms^34^: one investigating the single details of molecular combinations or regulatory networks, alike to “microstates” in statistical mechanics,^35^ and another looking for general analytical models, built upon kinetic theories, to understand complicated biochemical processes in simpler and general terms.^36^ The latter allows us to construct classes of systems according to universal routes of dynamical development, regardless of the microscopic details. We leverage this paradigm to make sense of critical transitions and identify the most relevant classes pertaining to biological systems. Box 1 provides such classification, comparing critical transition to smooth transitions, illustrating a connection between mathematical modeling and empirical observations in the field.^37^ In the rest of the article, we also provide guidance for EWS selection and optimization, depending on realistic noise properties and other notable features of classes of complex systems, developing new composite indicators.

Box 1 **Classification of critical transitions**

Consider a dynamical system whose state (or regime) is usefully characterized by a set of dynamic variables $x\in {\mathbb{R}}^{m}$, whose relations to each other are modeled by a set of parameters $p\in {\mathbb{R}}^{n}$:

(Equation 1) $$\frac{dx}{dt}=F\left(x\left(t\right),p\right)\text{,}$$

where $F:R^{n+m}\to R^{n}$ is a system of sufficiently smooth functions. If *p* is not explicitly dependent on time, the system is termed *autonomous*; if p=p(t), the system is called *non-autonomous*. The distinction between autonomous and non-autonomous can be supported when considering naturally fixed parameters,^38^ or when addressing timescale separation (“slow-fast system”) between biochemical processes, like mRNA transcription versus protein degradation times.^39^ This results in sets of dynamical (for variables) and algebraic (for parameters, termed at quasi-steady state) equations.^40^ Together, variables and parameters define and shape a state space (or “landscape”) that, if F(x,p) has elements of non-linearity, can be characterised by multiple attractors,^41^ i.e., region of stability for system’s states. If parameters are allowed to change (either non-autonomously, or at quasi-steady state), the state space is dynamic and attractors can change, as opposed to static landscapes like Waddington’s.

The state space can be multidimensional. However, near bifurcation points, it can be aptly described using low-dimensional models associated with critical thresholds in the values of leading parameters (usually corresponding to the largest eigenvalues^42^). Such models are termed “normal forms” of a dynamical system, simplified minimal-order forms that determine the system’s behavior and retain universal properties of generic bifurcations (see Kuehn et al.^43^ and STAR Methods). Normal forms can be inferred from bistability properties^14^ or deduced from network models, if they are available for the considered systems.^44^^,^^45^

In addition to bifurcation points, noise can characterize the system’s dynamics. Noise is ubiquitous in biology^46^^,^^47^ and can correspond to stochasticity in intrinsic biochemical processes or cell-cell variation.^48^ Mathematically, noise variables can be modeled as fast degrees of freedom augmenting system ((1)), which is a dualistic representation to stochastic processes.^49^ Noise can push the system out of original attractors onto new ones, therefore causing random switches between phenotypic states even in the absence of dynamical bifurcations.

We propose to use the relative timescales between dynamical variables, parameters and noise to develop a systematic classification of transitions between system states. This way, we synthesize and improve the contributions of Thompson et al.,^50^ Kuehn et al.,^19^ Ashwin et al.,^51^ Shi et al.^52^ toward the establishment of a theory on critical transitions in real systems. To do so, let us extend and disentangle Equation 1 to explicit the dependencies on state variables $x\in R^{m}$ and system parameters $p\in R^{n}$, on the introduced stochastic variables $\xi \in R^{l}$ and on the relative timescales modeled by time parameters $\tau_{i},i=\left\{x,p,\xi \right\}$. This results in a multiscale slow-fast system

(Equation 2) $$\left\{ \begin{array}{c} \tau_{x}\frac{dx}{dt}=f\left(x,p,\xi \right)\\ \tau_{p}\frac{dp}{dt}=g\left(x,p,\xi \right)\\ \tau_{\xi }\frac{d\xi }{dt}=h\left(x,p,\xi \right)\;\text{.} \end{array}\right.$$

Using this representation, tipping systems can be classified into three main classes of critical transitions on the basis of relative timescales: bifurcation-induced (“b-tipping”), noise-induced (“n-tipping”) and rate-induced (“r-tipping”), following the nomenclature introduced by Ashwin et al.^51^:

(Equation 3) $$\begin{array}{c} b-\text{tipping}:\tau_{p}\gg \tau_{x}\gg \tau_{\xi }\\ n-\text{tipping}:\tau_{p}\gg \tau_{x}≃\tau_{\xi }\\ r-\text{tipping}:\tau_{p}≃\tau_{x}\gg \tau_{\xi } \end{array}$$

If $\tau_{\xi }>\tau_{x}$, the system becomes ergodic and visits the full state-space uniformly without displaying transitions.^52^

The b-tipping class thus encompasses all those transitions primarily driven by bifurcations, i.e., slow changes in control mechanisms modeled as quasi-steady approaches of leading parameters to their threshold values. They modify the attractor landscape, in the presence of low noise-to-signal ratios, and can be further sub-classified according to dimension *m* and co-dimension *n*.^50^ In this work, we only consider low-dimensional ones, commonly found in cell dynamics studies. Examples include toggle-switch mechanisms for the lac-operon,^53^ population collapses of microbiological colonies past threshold concentrations of stressors or nutrients,^54^ or epithelial-mesenchymal determination.^55^ Higher *m* and *n* yield more complex bifurcations associated with, e.g., neural network activity.^56^

The n-tipping class groups various transitions driven by stochastic fluctuations on fixed landscapes, including large, impactful and unexpected events (sometimes called “dragon kings”^57^). Example range from enzymes crossing activation chemical barriers via “promoting vibrations”,^58^ “rebellious cells” undergoing contrasting development pathways during cell reprogramming,^17^ and other long-studied cases of noise-induced transitions.^59^

B-tipping and n-tipping directly link to the aforementioned debates in systems biology about deterministic or stochastic drivers of critical changes. R-tipping refers to critical ramping of control parameters, not coped by the system, which has been so far observed in climate^60^ and engineering^61^ systems. The heat-shock response of plants to ramping temperature conditions^62^ may fall within this class, but further studies are required. The critical transition classes can be visualized on bifurcation diagrams or using quasi-potential landscapes,^63^ which can be obtained as integrals of vector fields like Equation 1 or inferred from data.

**Figure 1:**
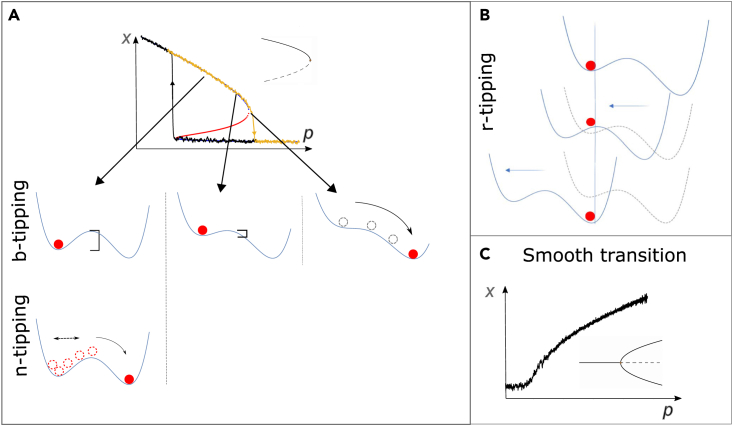
Classification and illustration of transitions between states *x* of a dynamical system *x* and *p* may also correspond to network combinations of observable variables and parameters.^37^^,^^44^ (A) Illustration of b-tipping and n-tipping using a bistable system with saddle-node bifurcations (unstable branch in red; saddle-node template shown in inset). Hysteresis can occur, i.e., asymmetric routes to tipping from one stable state or from the other (orange, from up to down with increasing *p*; black, from down to up with decreasing *p*). In b-tipping, the system (depicted as a red ball) approaches the bifurcation point. The associated landscape is molded by *p* and the basin of attraction becomes shallower (as visualized by the bars) until disappearing; there, the system tips. N-tipping: if subject to strong fluctuations (dashed arrow), the system “fluctuates” (dashed ball) and can overcome the barrier onto an alternative attractor, even if far from the bifurcation point. (B) Illustration of r-tipping using the landscape representation: rapid ramping of the control parameter makes it as if the landscape shifts and the systems does not manage to move along, therefore tipping onto another attractor “sliding” underneath. Here, hysteresis may not be present, e.g., if the landscape is symmetric. See Ashwin et al.^51^ for formal definitions. (C) For contrast, an example of “smooth” transition without hysteresis is provided using a dynamical system close to a pitchfork bifurcation (inset) as template. To reproduce the plots, see STAR Methods.

Figure 1 shows the classification between the transition classes, with illustrative examples of what can happen to systems within simplified attractors. Note that the hard-cut classification derives from the mathematical assumptions in Equation 3: gradients between the transition classes may exist and call for deep investigation. In particular, our work focuses on “noisy bifurcations”, i.e., dynamics characterized by bifurcation points and the presence of low to moderate noise-to-signal ratio.

Our work bridges mathematical insights and observations of real systems to classify various tipping mechanisms. There are ongoing debates whether regime shifts in biological systems, like cell-fate decision, are primarily driven by deterministic bifurcations^64^^,^^65^ or by random fluctuations,^35^^,^^66^ which prompted several authors to question the old “Waddington landscape” interpretation.^37^ By systematically analyzing known regime shifts, we classify the mathematical models to address various types of critical transitions, subject to combinations of bifurcations and noise,^49^ and to develop a method to extract systems’ robustness proxies from data. For clarity, this topic is covered in Box 2.

Box 2 **Bifurcations with noise and system robustness**

Among the critical transition classes described above, let us consider those primarily driven by bifurcations, with noise further influencing the dynamics. In this sense, we can speak of “noisy b-tipping”, with the first condition in Equation 3 becoming

(Equation 4) $$\tau_{p}\gg \tau_{x}>\tau_{\xi }\text{,}$$

that is, the noise-to-signal ratio is not negligible but the slow-fast condition between variables and parameters still applies.

**Figure 2:**
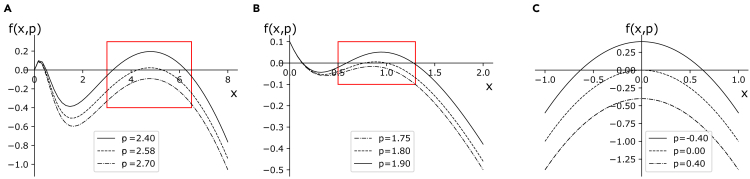
Visual example of topological equivalence (A) Plot for $dX/dt=f\left(x,p=c^{\prime }\right)=X\left(1-X/K\right)-c^{\prime }X^{2}/\left(X^{2}+1\right)$, a model of harvested ecological populations,^1^ also akin to Allee effects observed in microbiological colonies^26^; *X* is the population density, *K* is the carrying capacity and $c^{\prime }$ is the maximum harvest rate. (B) Plot for f(x,c) of the autocatalytic loop model Eq. 15. (C) Plot for f(x,p) of the saddle node normal form $\dot{x}=-p-x^{2}$. The two realistic models are locally topologically equivalent to the normal form within the red rectangle (visual reference): they approach a bifurcation point, marked by f(x,p) crossing the x axis, as the parameter $c^{\prime }$ or *c* changes.

For this class, normal forms can be used to analytically study systems’ robustness and derive EWS for impending tipping points.^19^ Normal forms are general and low-dimensional models $\dot{x}=f\left(x,p\right)$ that describe topologically equivalent systems within a bifurcation class, in the vicinity of critical points.^42^ They allow to extract analytical and generic results for wide classes of systems,^43^ at the price of neglecting homeostatic dynamics far from tipping points. As a result, they allow to focus on critical transition mechanisms across various systems, instead of studying the full evolution of a single system. Details about topological equivalence and construction of normal forms are in STAR Methods. Figure 2 shows an example of reduction to normal forms for two simple models.

Here, we consider those normal forms of primary biological interest. The saddle-node bifurcation, often associated with population collapses^1^^,^^26^ or biological state transitions,^67^ is defined by $f\left(x,p\right)=\pm p\pm x^{2}$. At p=0, a stable (${\tilde{x}}_{s}=\sqrt{p}$) and unstable $\left({\tilde{x}}_{u}=-\sqrt{p}\right)$ branch collide and vanish, resulting in a critical transition to an alternative branch (if it exists). Transcritical bifurcations $f\left(x,p\right)=px-x^{2}$ are characteristic, for instance, of epidemic outbreaks.^28^ Here, the two equilibria $x_{1}=0$ and $x_{2}=p$ meet at p=0 and exchange stability. Finally, the family of pitchfork bifurcations $f\left(x,p\right)=px+l\;x^{3}$ describe branching processes from one to two states (or vice versa); l>0 identifies subcritical bifurcations, associated with critical transitions, while l<0 defines the supercritical case, with a continuous transition over mean values. This mechanism is identified in cell regulation processes.^37^

Stochastically forced systems, associated with “noisy b-tipping”, can be written in the Itô form^50^

(Equation 5) dx=f(x,p)dt+h(x,p)dW,

where dW is a Wiener process with variance σ and f(x,p) is a suitable normal form from those described above. The term h(x,p) allows us to represent different noise types, to reflect modern knowledge of stochastic processes occurring in biological systems. Additive Gaussian noise with h(x,p)=1 is usually associated with extrinsic cell-cell variability. State-dependent (multiplicative) noise h(x,p)≠const represents intrinsic stochasticity determined by, e.g., reaction rates, timescales or species concentrations of the underlying biochemical processes.^68^ Combinations of additive and multiplicative noise, with various ratios depending on different systems, are more realistic^69^^,^^70^ and fit experimental data better than Gaussian noise.^71^ Another interesting case is colored (time-dependent) noise^32^^,^^72^; however, combining additive and multiplicative noise processes is usually considered a valid modeling alternative to reproduce biological stochastic dynamics.^69^ Hence, this paper focuses on the noise combinations, while the reader is referred to the previous publications for the specific case of colored noise. If the microscopic kinetics is known, the noise terms can be exactly derived from the Master equation using Gillespie formalism.^73^ Alternatively, a diffusion approximation^74^^,^^75^ derives noise terms proportional to system state (h(x,p)=x), or to the drift term of Equation 5, h(x,p)∝f(x,p). Here, for multiplicative noise, we consider $h\left(x,p\right)=\sqrt{f\left(x,p\right)}$^68^ and h(x,p)=f(x,p), to reflect modeling of biological regulatory circuits.^76^ This way, mechanistic and stochastic normal-form bifurcation models are examined to study the effects of intrinsic and extrinsic noise on statistical patterns of variability and related EWS.

Following the procedure detailed in STAR Methods, Equation 5 is analyzed by solving the slow dynamics, linearizing around a trajectory inside the stable (attracting) manifold and changing the coordinates to highlight the residuals y(t) around the linearization. This procedure gives

(Equation 6) $$dy=\partial_{x}f\left({\tilde{x}}_{s}\left(t\right),t\right)y\;dt+\sqrt{h^{2}\left(x\right)}dW$$

where ${\tilde{x}}_{s}$ corresponds to the attracting part of the critical manifold (stable solutions). The linearised drift term corresponds to the leading eigenvalue of the deterministic normal form. Its magnitude is the asymptotic decay rate of a perturbation. It corresponds to the concept of engineering resilience,^77^ which is akin to that of robustness.^78^ A change of notation $\left|\partial_{x}f\left({\tilde{x}}_{s}\left(t\right),t\right)\right|=k$ makes explicit that Equation 6 corresponds to a (possibly non-autonomous) Ornstein-Uhlenbeck process, with critical *k* given by $k_{0}=0$. It is a well-studied problem in stochastic processes theory, with analytical solutions for its statistics in different regimes.^74^^,^^79^ Equation 6 can be regarded as a first order autoregressive model. However, its derivation from normal forms allows more nuanced interpretation: rather than being hypothesized as a statistical model to capture simple relationships, it is general for all models that can be reduced to normal forms.

For the subsequent analysis of EWS in settings that are typically observed in systems biology studies, we first employ a framework based on dynamical manifolds, underpinning universal routes to explosive transitions.^43^ This characterizes the warning signals associated with “noisy” bifurcations, and studies their dependency on noise properties and other dynamical features like rapid approaches to threshold values. This way, we provide general results about EWS robustness and sensitivity to dynamical features, to guide applications on various systems, understand their limitations and promote future developments.

Then, we focus on a critical transition sub-class of high biological relevance, the stochastic saddle-node bifurcation.^36^ For this tractable, yet realistic model of complex biological processes, we develop a composite EWS indicator to optimize the leading time of the alerts, i.e., how much in advance reliable signals are triggered, with respect to an impending transition. The new indicator is optimized over realistic noise types using the common genetic toggle switch model,^15^ as representative of the considered CT class. This way, we overcome the limitations of other EWS from literature, which have mostly been developed over Gaussian noise whilst biological systems usually feature correlated and state-dependent noise.^48^^,^^76^^,^^80^ Thanks to this extension, the indicator also provides additional insights about the systems under investigation, such as inference of noise type from data. The theoretical results are finally tested and verified on publicly available experimental data, demonstrating their potential for monitoring and interpreting diverse systems.

## Results

### Robustness of EWS for noisy bifurcations

Within the class of critical transitions induced by bifurcations characterized by small fluctuations, discussed in Box 1 and 2, we study the EWS associated with impending tipping points, considering different noise types that are better representative of biological dynamics that additive Gaussian noise (see Box 2). Albeit any type of multiplicative noise can in principle be considered, we focus on the cases h(x,p)=x, $h\left(x,p\right)=\sqrt{f\left(x,p\right)}$ and h(x,p)=f(x,p) as the most commonly employed in the field, see Box 2 for explanation.

Analytic expressions for key summary statistics indicators can be obtained from Equation 6 using standard approaches for stochastic processes.^74^^,^^79^ Their behavior as the control parameter changes provides EWS for approaching noisy bifurcations.^1^ The lag-τ autocorrelation function does not depend on $h^{2}\left(x,p\right)$ but only on $\left|\partial_{x}f\left({\tilde{x}}_{s},p\right)\right|=k$:

(Equation 7) $$AC\left(\tau \right)=e^{-k\tau }\;\text{.}$$

Hence, the common indicator lag-1 autocorrelation (AC(1), with τ=1) only depends on the dampening rate. The power spectrum of the Fourier transforms and the variance, two common indicators, explicitly depend on $h^{2}\left(x,\;p\right)$:

(Equation 8) $$S\left(\omega \right)=\frac{h^{2}\left(\tilde{x},p\right)}{k^{2}+\omega^{2}}$$

(Equation 9) $$\text{Var}=\frac{h^{2}\left(\tilde{x},p\right)}{2k}\;\text{.}$$

As discussed by Bury et al.,^81^ the power spectrum can provide an increasing signal over time-series data thanks to its quadratic scaling $\propto k^{-2}$, but it requires high sampling frequency that is often not available. Coefficient of variation (CV) and Index of dispersion (ID), defined as

(Equation 10) $$CV=\frac{\sqrt{\text{Var}}}{{\tilde{x}}_{s}},ID=\frac{\text{Var}}{{\tilde{x}}_{s}}\text{,}$$

also depend on $h^{2}\left(\tilde{x},p\right)$. Other statistical moments, for stochastic processes with quasi-steady state parameter, can be expressed as

(Equation 11) $$\left\langle y^{\nu }\right\rangle -{\left\langle y\right\rangle }^{\nu }=\int_{-\infty }^{\infty }{\left(y^{\prime }-\mu \right)}^{\nu }P\left(y^{\prime }\right)dy^{\prime }$$

where $P\left(y^{\prime }\right)$ is the probability density function from the associated Fokker-Plank equation^79^ and μ is the expected average value. Skewness and kurtosis, sometimes suggested as indicators for EWS,^82^ can be easily extracted from Equation 11 as third and fourth moments (ν=3 and 4). Entropy-based indicators are more challenging to derive in case of multiplicative noise, as their defining integrals may not be solvable. Their derivation in case of Gaussian noise is described in STAR Methods; for the other cases, their behavior is estimated below using computer simulations.

**Figure 3:**
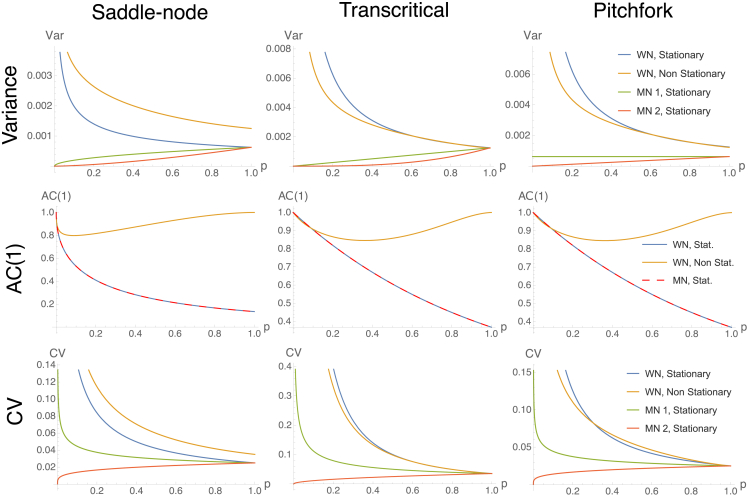
Trends of common statistical indicators We consider Var, AC(1) and CV for saddle-node, transcritical and pitchfork bifurcations as p→0, in different dynamical contexts (combinations of noise characteristics and stationarity for the control parameter). WN: additive white (Gaussian) noise; MN 1: mult noise with $h\left(\tilde{x},p\right)=x$; MN 2: mult. noise with $h\left(\tilde{x},p\right)=x^{2}$. As the autocorrelation is independent on noise, only MN 1 is show and it overlaps with the additive noise case.

In all cases, the analytical results for each normal form can be obtained by substituting the corresponding dependency of the drift term to the control parameter: for the saddle-node, $k=2\sqrt{p}$, for the transcritical k=p and for the pitchforks k=2p. In Figure 3, the effect of multiplicative noise on the trends of common indicators is shown using $h\left(\tilde{x},p\right)=x$ and $h\left(\tilde{x},p\right)=x^{2}$.

Figure 3 shows expected trends of common statistical indicators, for the three main normal forms and different noise types. The figure is derived by solving the equations above and substituting the corresponding noise functions $h^{2}\left(x,p\right)$. Although the scaling induced by differs, the qualitative trends are conserved across the bifurcations. This observation suggests genericity of EWS, but also difficulties to infer the existence of one or another bifurcation using statistical indicators alone (see also Kefi et al.,^83^ Boettiger et al.^84^). Other methods (e.g., Angeli et al.^14^) are recommended to complement the inference.

For Gaussian noise, EWS are associated with increasing trends of statistical indicators.^1^^,^^85^ However, multiplicative noise may alter or completely disrupt them (as also noted by O’Regan et al.^68^), resulting in no early warnings prior to tipping points. Equation 8 shows that even power spectrum trends can be subject to alterations from expected patterns, potentially resulting in spurious signals.

A preliminary investigation on ramping parameters^86^ can also be conducted. In this case, $\tau_{x}≃\tau_{p}$: the quasi-steady-state (stationary) assumption is relaxed, but r-tipping may not yet occur. Let us consider linear ramping as $k=k_{0}-at$, where $k_{0}$ is any initial condition, *a* is a small rate coefficient and the ramping stops at the critical value k=0. Both coefficients are set to 1 to represent commensurable time scales. Only Gaussian noise is considered. This is a particular case of inhomogeneous processes^79^ for which statistical moment solutions exist in the form

(Equation 12) $$\left\langle y\left(t\right)\right\rangle =e^{-\int_{0}^{t}k\left(t^{\prime }\right)dt^{\prime }}$$

(Equation 13) $$\left\langle y\left(t\right)y\left(t^{\prime }\right)\right\rangle =\frac{\sigma^{2}}{2k}e^{-2\int_{0}^{t}k\left(t^{\prime \prime }\right)dt^{\prime \prime }}+\sigma^{2}\int_{0}^{t}e^{-2\int_{t^{\prime }}^{t}k\left(s\right)ds}dt^{\prime }\;\text{.}$$

Derived statistics are calculated analogously. Equation 13 is solved using Mathematica software to tackle the rightmost integral yielding the non-elementary Error function Erf(t). Figure 3 shows that trends of common indicators may be modified by commensurable time scales of parameters evolution. Hence, raising reliable alerts becomes more challenging.

Overall, this analysis demonstrates that theoretical EWS because of increasing trends of summary statistics are sensitive to the “dynamical context”, i.e., noise properties and reciprocal time-scales. Hence, if the dynamical context is not carefully accounted for, spurious signals may be extracted from data, as observed in early findings from single systems.^28^^,^^87^

If the context is known, the current results suggest which indicators to use to obtain robust early warnings. The autocorrelation is robust to whether noise is additive or multiplicative; the variance is more sensitive to multiplicative noise, but maintains its expected trends in case of ramping parameters. The coefficient of variation, calculated by using its definition Equation 10 together with Equation 13, and solving it using the Mathematica software, is also robust in case of commensurable time scales and copes well in case of certain types of multiplicative noise. Overall, what matters is the competition between changes in noise and changes in resilience: depending on which one is more rapid, the indicators and their associated EWS may perform as expected or fail to anticipate an impending critical transition.

Measurement processes or details of realistic models may further influence EWS. Measurement uncertainties, assumed as Poisson processes associated with measuring instruments or procedures and thus independent of systems’ dynamics, can be introduced in the formulas of statistical indicators by error propagation in quadrature (see STAR Methods for details). In case of Gaussian noise and stationary processes, the expected trends of common indicators are not altered, hence, EWS can be in principle extracted even when using noisy measurements (*cf.* STAR Methods).

Single indicators may also be skewed in case realistic details are considered. For instance, on empirical data, normalizing by the critical value and set a normal form around $p_{0}=0$ and ${\tilde{x}}_{s}\left(p\right)=0$ may be challenging, since such critical values are largely unknown. Hence, instead of computing ${\tilde{x}}_{s}\left(p\right)\to 0$ like on perfectly reconstructed normal forms, ${\tilde{x}}_{s}\left(p\right)\to x_{0}^{\prime }$ is often computed,^54^ where $x_{0}^{\prime }$ corresponds to the critical value, unknown *a priori*. Such case can be modeled as ${\tilde{x}}_{s}\left(p\right)=x_{0}^{\prime }+\sqrt{p}$. Hence, Equation 10 becomes

(Equation 14) $$CV_{r}=\frac{\sqrt{\text{Var}}}{x_{0}^{\prime }+\sqrt{p}}\;\text{.}$$

Here, other multiplicative noise forms may alter its behavior and shadow possible early warnings. Finally, skewness and kurtosis calculated from Equation 11 display increasing trends when $P\left(y^{\prime }\right)$ is symmetric (STAR Methods). However, this may not be true in case of multiplicative noise,^15^ resulting in distorted trends and early warnings. In this sense, there is no ambiguity between the results of Guttal et al.,^82^ proposing EWS from skewness, and Dai et al.,^26^ observing flat and fluctuating trends on experimental data: likely, the noise properties were different than what assumed.

### Optimization of EWS

Having assessed in which cases the proposed EWS are expected to work for noisy b-tipping transitions, we now optimize their performance to provide significant and as-early-*as*-possible alerts, in a range of dynamical contexts and for the most common transitions observed in systems biology. To this end, we focus on multistable systems,^55^ develop and solve an optimization problem using computer simulations to go beyond the first-order approximation from Equation 6 (see STAR Methods for details), and study a wide range of noise levels and types, to establish a composite indicator that is robust and performing across multiple systems.

**Figure 4:**
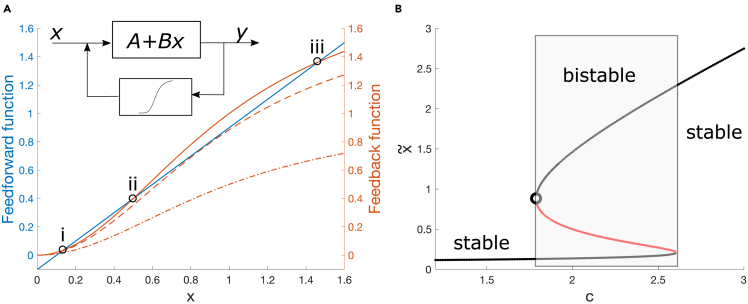
Bistable systems, studied with (a) characteristic curves or (b) bifurcation diagram $\tilde{x}$ (stable state) versus control parameter Among the systems undergoing saddle-node bifurcations, any linear system with nonlinear feedback and adequate feedback gain, such that the characteristic curve crosses the activation function in three points (two stable, one unstable), can display bistability. This example uses Eq. 15. (A and B) The feedback function FB corresponds to the Hill function (k=2), the feedforward FF to the linear part −(K−x) with *K* in its appropriate range. The control parameter *c* tunes the FB function. Dashed-dotted line: *c* is not sufficient to promote bistability, corresponding to left stable region of (b). Dashed line: the critical value for which FB is tangent to FF, corresponding to saddle-node point, open circle in (b). Solid line: bistable system with three intersection points (stable, i and iii; unstable, ii). When studying the vector field f(x,c) is easier than the characteristic curves, one can use the representation and interpretation in Figure 2B. Note that the line styles have the same meaning in panel (a) and Figure 2.

Multistable systems are systems whose deterministic landscape features at least two attractors,^88^ and usually undergo either saddle-node bifurcations or n-tipping. Bistability means local multistability across two attractors. Angeli et al.^14^ provide necessary and sufficient conditions for bistability in a wide range of biological systems. Among them, a feedback model with three-points I/O characteristic curves suffices. A simple linear system with monotonic sigmoidal feedback can do the job, in a range of parameters (Figure 4). As a case study, the autocatalytic positive feedback loop derived from Michelis-Menten kynetics^15^

(Equation 15) $$\dot{x}=f\left(x,c\right)+\eta \left(t\right)=K+c\frac{x^{k}}{1+x^{k}}-x+\eta \left(t\right)\;\text{.}$$

satisfies the bistability conditions, and can thus display transitions between attractors, if $0<K<1/\left(3\sqrt{3}\right)$ for k=2.^89^ In Equation 15, *x* is the concentration of a transcriptional factor activator, activating its own transcriptions when bound to a responsive element; *K* is the basal expression rate, *c* is the maximum production rate, *k* is the Hill coefficient and η(t) accounts for the stochastic terms. Equation 15 comes from a two-variable genetic toggle switch, assuming slow-fast timescale separation between the two variables^90^ and after a-dimensionalizing the chemical details to retain the dynamical scaffold. Notably, networks of Michelis-Menten regulators can be reduced to Equation 15 after dimension reduction techniques.^44^

Equation 15 displays bistability for a range of values *c* (the exact range depends on *K* and *k*^91^) and, in particular, a saddle-node bifurcation between two alternative steady states at a critical value $c_{0}$ of the parameter *c*, such that $\partial f/\partial x{|}_{\left(\tilde{x},c_{0}\right)}=0$:

(Equation 16) $$c_{0}=\frac{{\left(x_{0}^{k}+1\right)}^{2}}{kx_{0}^{k-1}}$$

where $x_{0}$ is the tipping value for the system state. Therefore, system (15) can be used as a paradigmatic example of biological systems, within the saddle-node b-tipping class, to perform optimization studies that go beyond the local and low-noise-to-signal-ratio approximation provided by normal forms.

The quasi-steady state assumption is generally accepted for such systems,^40^ so we do not consider the case of commensurable time scales but we focus on dynamical contexts characterized by different types of noise, whether yielding n-tipping or possibly skewing statistical indicators because of multiplicative and/or additive nature. To model combinations of intrinsic and extrinsic noise, we set

(Equation 17) η(t)=[α+(1−α)h(x)]dW,

where α weights the additive or multiplicative noise component (α=1 corresponds to additive Gaussian noise, α=0 to solely multiplicative); like above, h(x)=x or $h\left(x\right)\propto f\left(x\right)=x^{k}/\left(1+x^{k}\right)$^76^ and dW is a Wiener process with variance σ. Without loss of generality,^91^ we set k=2.

**Figure 5:**
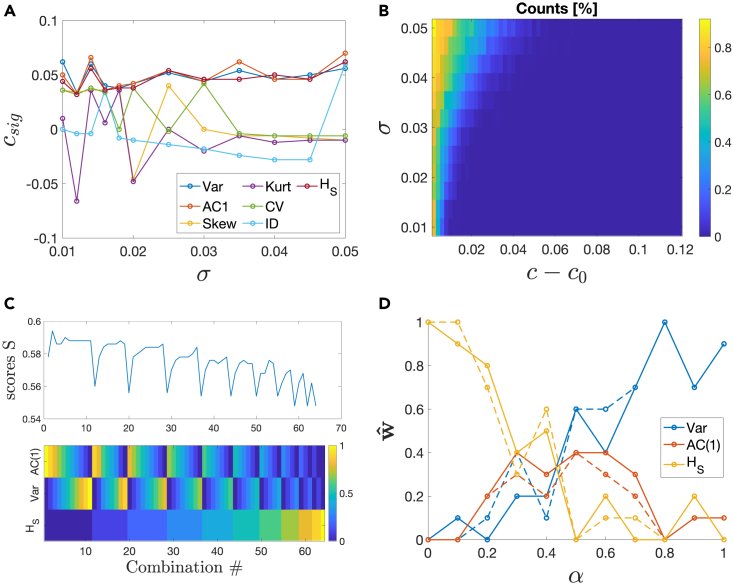
Optimization of leading indicators for EWS, according to lead parameter $c_{sig}$ (A) $c_{sig}$ at various noise intensities σ, for all the most common indicators. (B) The counter C, normalised by all transitions to be interpreted as probability of n-tipping, at different noise intensities σ and distances $c-c_{0}$ from the bifurcation point. (C) Scores S, corresponding to the argument of the cost function Equation 19, for various combinations S from Equation 18. In the panel below, the color code shows the weights $w_{k}$ for each indicator, in each combination. Results in panels a, b and c refer to α=1. For various types of h(x) and α=0, see Supplementary Figure S3. (D) Optimal weights $\hat{w}$ for each indicator, as a function of noise mixing α. As a representative of multiplicative noise, we used h(x)=x. Other h(x) conserve the trends, albeit changing the corresponding $c_{sig}$. It may happen that the optimization is solved by multiple combinations (dashed lines).

As EWS are associated with increases of statistical indicators, we need to establish a measure of statistically significant increase, to rule out false positives and false negatives because of random fluctuations in the indicators. To do so, we employ the p value analysis used in Proverbio et al.^91^ (see STAR Methods for details). It allows us to measure at which value of the control parameter *c*, before $c_{0}$, a significant signal is triggered, thus obtaining a “lead-parameter” $c_{sig}^{I}\left(\sigma ,\alpha \right)$ depending on noise properties and the considered indicator I (see STAR Methods for details). $c_{sig}^{I}\left(\sigma ,\alpha \right)$ is first computed for each indicator individually. Figure 5A shows the results in case of additive noise, while various functionals of multiplicative noise h(x) (with α=0) are reported in Figure S3. Each indicator yields various $c_{sig}$; in Figure 5A, Var, AC(1) and $H_{S}$ maximize $c_{sig}$ over various noise levels, while other indicators like skewness and kurtosis perform poorly, as anticipated by the analytical results. CV and ID are also rather poor, likely because of fluctuations of mean values and anticipating n-tipping (*cf.* also Figures S2 and S3). For the case of multiplicative noise (Figure S3), $H_{S}$ keeps performing well while Var, as expected from the theoretical analysis, decreases its performance despite being still better than Skew and Kurt.

Complementing the analysis of the lead parameter requires understanding how many noise-induced tipping events occurred before it and assessing whether the increasing indicators alert for impending collapses or reflect transitions that have already happened. The analysis thus interprets warning indicators as “anticipating” or “just-on-time detecting” the tipping events. To do so, a counter C quantifies, for each parameter value *c* and for each noise level σ, how many trajectories tip onto the alternative stable state. The results are in Figure 5B: as σ increases, more n-tipping events occur before the bifurcation point. In particular for σ>0.42, several noise-induced transitions occur at $c≃c_{sig}$. Hence, as noise increases, the indicators capture ongoing critical transitions but are not able anymore to provide much earlier alerts. This likely explains the remarks from Dudney et al.,^31^ that EWS could not anticipate several transitions in real-world systems, in particular those characterized by high noise-to-signal ratios.

The previous results are also employed to define an optimization problem to maximize $c_{sig}^{I}\left(\sigma ,\alpha \right)$ for varying α. To do so, we define a composite indicator as linear combination of indicators

(Equation 18) $$S=\sum_{k}w_{k}I_{k}$$

and look for a set of weights $w=\left\{w_{k}\right\}$ that maximizes all $c_{sig}^{I}\left(\sigma ,\alpha \right)$ as σ increases (to guarantee robustness against noise levels), for the various α:

(Equation 19) $$\hat{w}\;s.t.\;\max_{w}S=\max_{w}\left[\sum_{l}c_{sig}^{S}\left(w,\sigma_{l}\right)\right]\text{,}$$

where S are scores composed by sums of $c_{sig}^{S}\left(w,\sigma_{l}\right)$ over all σ. In the set I, we include those indicators that are expected to be robust and performing, first and foremost in the additive noise case. Leveraging on the previous results, we therefore select Var, AC(1) and $H_{S}$. As the problem is non-convex (Figure 5C), we perform a grid search for all combinations of $w_{k}$, with a stride 0.1 and such that $\sum_{k}w_{k}=1$. See Fig. Figure 5C for the considered combinations to construct S.

Figure 5D reports the results of the optimization procedure. Combinations of Var and AC(1) make up for optimal indicators in case of additive noise, $\hat{w}=\left[0.9,0.1,0\right]$ for Var, AC(1) and $H_{S}$, respectively, in case of α=0. In this case, $H_{S}$ is log-proportional to Var (see Equation 30) and does not add much information. In turn, combining the indicators maximizes $c_{sig}^{S}\left(\sigma ,\alpha \right)$ in case of mixed noise types. Finally, when multiplicative noise is prevalent in the system, using Shannon entropy is preferred ($\hat{w}=\left[0,0,1\right]$ for α=0). Note that, as the problem is non-convex, there may be more than one combination to create the optimal S. However, changes in weights $w_{k}$ are always within $\Delta w_{k}∼\pm 10\%w_{k}$ and the trends are conserved (see dashed lines in Figure 5D). By following the computational procedure to perform a sensitivity analysis, we observe that such small $\Delta w_{k}$ yield changes of ±4% on the scores S, on average over all α (ΔS∈[1.8;6.5]%), while off-setting $w_{k}$ by more than 50% (e.g., using full variance in case of multiplicative noise) worsens S (and consequently the optimal lead parameter) up to more than 20%.

### Verification on experimental data

The theoretical predictions are verified and used to interpret experimental data from a previous publication.^26^ The data are sampled from controlled experiments of budding yeast population collapse. Budding yeast cooperatively breaks down the sucrose necessary for its survival, thus inducing a density-dependent dynamics that realizes the Allee effect of bistable population dynamics (*cf.* Figure 2B). Repeated experiments empirically reproduced a saddle-node bifurcation by measuring population density (state variable) as a function of dilution factors (DF, control parameters) affecting the sucrose environment. Various EWS for population collapse can be estimated using distribution data. More details about data collection and analysis are in STAR Methods. Testing our theoretical results on a different system than Equation 15, yet still belonging to the saddle-node driven b-tipping class, would thus assess their generic applicability within this class.

**Figure 6:**
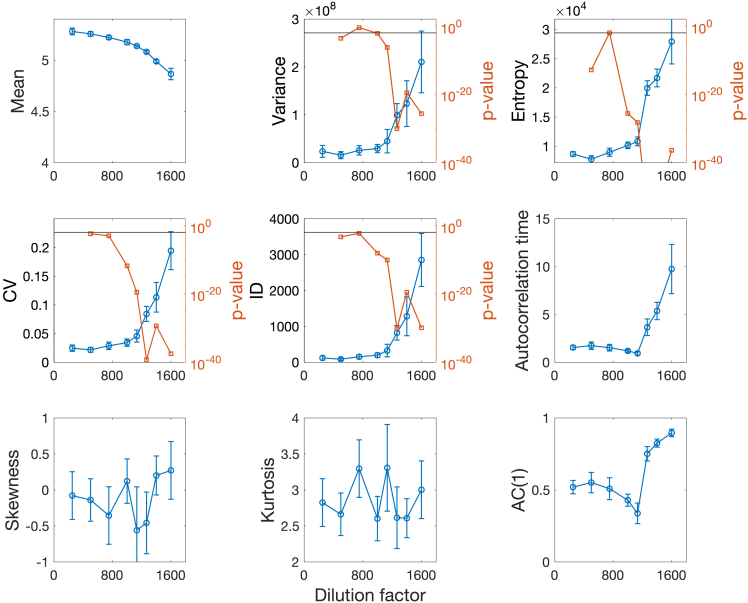
Statistical indicators calculated on empirical data Data are from Dai et al.,^26^ as functions of dilution factor (DF). Their corresponding p values are estimated when the trend is increasing while approaching the bifurcation point (rightwards point). All statistical moments of degree γ have units of measure ${\left(cells/\mu l\right)}^{\gamma }$. The autocorrelation time is in days. The mean reproduces the upper stable branch of a saddle-node bifurcation diagram (*cf.*Figure 1) until the empirically estimated bifurcation point at DF = 1600. Horizontal solid lines mark p value = 0.01. Error bars are obtained from bootstrapping of the original data. The p-values are calculated for distribution data with respect to the first distribution (baseline). For details, refer to STAR Methods.

Figure 6 shows trends of each indicator individually, as function of the dilution factor (with critical value at 1600). The error bars are estimated from bootstrapping (STAR Methods). Figure 6 reproduces the results from Dai et al.^26^ and includes the additional indicators considered in this paper. The mean is used to reconstruct the upper stable branch of a saddle-node bifurcation diagram (see Figure 1), reconstructed from data (the full diagram can be found in the original publication). However, it cannot be used as proper EWS as decreasing mean values could signify smooth changes rather than critical transitions, if the transition type and critical parameter are not known. Skewness and kurtosis fluctuate around 0 and 3, respectively, without providing EWS, as one expects in case of symmetric potentials (see Equation 36 and 0.18). AC(1) and the autocorrelation time (defined as −1/log[AC(1)]^26^) first drop before increasing sharply just before the critical value. Comparing it with Figure 3, we speculate that there are commensurable time scales between the intake of sugar by yeast cells and their evolution in density. Further experiments are suggested to check for this intriguing hypothesis.

Even in this case, as expected, Var, Entropy ($H_{S}$), CV and ID display monotonous increasing trends close to the bifurcation point. The increases are thus assessed using the p value test (*cf.* STAR Methods) to check whether they are significant or associated with fluctuations. To trigger a significant early warning signal, we require a conservatory significant p value <0.01. This way, we estimate the significant dilution factor ${DF}_{sig}$ for each indicator. For variance, ${DF}_{sig}=1133$, for the others DF. Comparing with the optimization results (from the previous section and Figure S3), we infer the presence of multiplicative noise in the system’s dynamics. Note that entropy showcases the smallest p value at ${DF}_{sig}=1000$; it is also the most robust when changing the repetitions in the bootstrapping procedure (STAR Methods).

**Figure 7:**
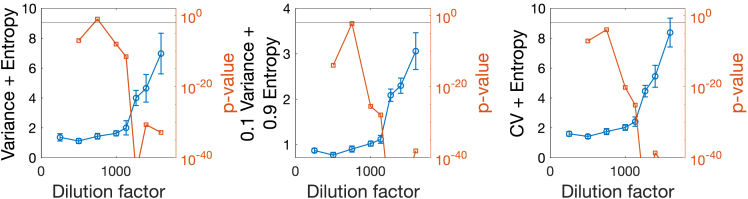
Combined indicators calculated on empirical data The analysis is analogous to that in Figure 6, using combinations of indicators. The horizontal solid lines mark p value = 0.01.

To test the hypothesis of association between EWS performance and noise type, we test combined indicators with $H_{S}$ and Var. According to the optimization above, the higher the variance content in the mixture, the lower the significance of the increasing trend. This is what is observed in Figure 7: having a balance between Var and $H_{S}$ yields ${DF}_{sig}=1000$, but with a higher p value than when reducing the ratio Var/ $H_{S}$ or when comparing with the case of entropy alone (from Figure 6).

Finally, we test combining CV and, since Figure 6 suggests that CV could perform well. Indeed, the new combined indicator yields ${DF}_{sig}=750$ (Figure 7, right), one dilution step before the others. This is not in contrast with the optimization analysis: CV is, in fact, expected to be as performing as $H_{S}$ if the noise levels are relatively high (see Figure S3). We recall that CV was not included in the optimization analysis to be generic and robust across noise types and levels. However, if high σ in state-dependent noise is known, constructing a composite indicator using both CV and $H_{S}$ may improve the alerting performance.

## Discussion

The paper provides a systematic classification of tipping mechanisms, highlights their underlying modeling assumptions, and bridges mathematical insights and observations of real biological systems to classify various tipping mechanisms, toward quantitative understanding and prediction of such relevant phenomena. The work shifts the focus from studying specific systems, that may undergo some transitions, to studying transitions, along with their classes and properties, which can accommodate the behavior of different systems. An interesting question for future studies will be to develop data-driven methods to classify each system within its corresponding class, much like those developed to distinguish stochastic or chaotic signals.^92^ This will dramatically help the understanding of biological processes and guide the selection of EWS or other methods to anticipate critical transitions, as well as informing methods to reconstruct cell developmental trajectories like those proposed by Eugenio et al.^93^

Moreover, we systematically investigate EWS associated with noisy bifurcation-induced transitions, key dynamical routes for the regulation and control of many natural processes. So far, EWS have been mostly studied in highly controlled computational settings, or checked on empirical data with alternate success. Our results make sense of previous observations, help to define their range of applicability to reliably predict systems’ behaviors, and allow to understand why spurious signals may be triggered in certain cases. We also assess whether and when EWS can be interpreted as anticipating or just-on-time detecting critical transitions in the presence of noise. By carefully analyzing noise types and parameter dynamics, we also extend previous results to more realistic settings, to guide real-world applications.

Using both analytical and computational methods, we observe that the variance – a highly employed indicator for EWS – may be sensitive to state-dependent noise, while AC(1) can be skewed by ramping control parameters. Both are good indicators in case of quasi-steady-state dynamics and Gaussian noise, with the ability to provide information about augmented risk of tipping events. In the other cases, Shannon entropy is the most robust and performing indicator and is suggested for applications in case of uncertain settings. If precise information about noise type and intensity are available, constructing composite indicators can improve the early-alerting performance, e.g., by combining CV and $H_{S}$.

After preliminary suggestions by Drake et al.,^20^ composite indicators have been subsequently tested, mostly based on system-specific traits.^94^ Contrarily, this paper introduces, demonstrates and optimizes a novel indicator based on summary statistics from normal forms, in principle extendable to numerous systems sharing similar dynamical features and noise properties. The optimization of composite indicators further points to the use of machine learning methods when abundant data are available,^95^ but also opens important caveats for their application in real life: feature combinations may be optimized for certain settings (e.g., noise intensity or type) but may be hardly generalizable for others. Our results remark that training should be performed considering all possible combinations, or by first assessing which critical transition class is being considering. Otherwise, misleading signals may be triggered and wrong conclusions reached. On the other hand, our results can be used for feature selection of more interpretable machine learning algorithms that leverage the proposed composite indicators, insofar defined for *a-priori* assessment of systems that lack big data.

This work provides results and guidelines for the application of EWS from the critical transitions framework, but some points should still be covered by future studies. They include more refined analytical derivations of indicators in case of inhomogeneous processes as well as closed formulae for entropy in exotic settings. Further investigations on realistic systems, including non-autonomous transitions currently understudied in systems biology, are thus suggested as extensions of our work. Similarly to the case of univariate indicators, which may fail to distinguish critical and non-critical transitions,^83^^,^^84^ multivariate indicators may be limited in capturing such differences. Other methods (e.g., Angeli et al.^14^) may be used to complement the inference, but further studies are suggested toward the inference of transition types using EWS. Another limitation of the present study is the restriction to low dimensional systems. In principle, they are representative of any system after dimension reduction techniques are applied (see e.g. Gao et al.^44^ and Laurence et al.,^96^ or Heino et al.^97^ for Principal Component Analysis-based techniques), but it is necessary to assess if and how the latter induce performance drops. Extending the analysis to high dimensional systems, e.g., by testing multivariate indicators^98^ or further refining EWS performance when multiple independent variables can be observed, is thus suggested to future studies. Moreover, our theoretical results have been verified on empirical data from literature, but we acknowledge the need of performing additional experiments to continuously validate our predictions. In particular, we suggest to design new experiments to test the quantitative predictions about lead parameters and to assess what happens in case of rapidly ramping parameters. Finally, although our results are in principle extendable to other fields, additional testing and validation is necessary if the data sampling differs and the ergodic equivalence between distribution data and time series may be challenged, as it is the case, e.g., for ecology or climate. Future targeted studies, that are beyond the scope of the current article, will likely cover this point, e.g. on data from Dakos et al.^99^ or Chen et al.^100^

Our results can be readily tested and applied on real-world monitoring systems and can inform the development of new indicators to address specific problems like cancer onset, much like previous studies^22^ did using less performing measurements. Our results can be further applied to a range of other diseases to detect their onset, from diabetes^101^ and other complex diseases^22^ to epidemics.^28^ In addition, leveraging the sensitivity of indicators’ trends to noise type and parameter dynamics can provide new methods to infer the latter from empirical data. For instance, comparing Figure 6 with Figure 3 supports hypothesis of commensurable time scales between intake of sucrose (affected by the dilution factor) and cells’ growth in yeast experiments^26^; such hypothesis, to be confirmed using controlled experiments and further computational studies, could advance our knowledge beyond the current slow-fast approximations.^40^ Similarly, the prevalence of certain noise types can be inferred by comparing data and theory. Overall, we connect theory and data, such that knowledge about the dynamical settings allows optimizing EWS, and analysis of statistical indicators enables inference of dynamical properties.

## STAR★Methods

### Key resources table

**Table:** 

REAGENT or RESOURCE	SOURCE	IDENTIFIER
**Deposited data**		

Budding yeast density	Dai et al.^26^	https://doi.org/10.5061/dryad.p2481134

**Software and algorithms**		

Matlab R2021b	Mathworks	https://www.mathworks.com/products/matlab.html
Mathematica v12	Wolfram	https://www.wolfram.com/mathematica/
Custom code	This paper	https://doi.org/10.5281/zenodo.7844650

### Resource availability

#### Lead contact

Further information and requests for resources and reagents should be directed to and will be fulfilled by Daniele Proverbio (daniele.proverbio@uni.lu)

#### Materials availability

This study did not generate new unique reagents.

### Method details

#### Topological equivalence and normal forms

Bifurcations model drastic changes in the qualitative behaviour of dynamical systems, such as shifts in equilibria and regimes.^42^^,^^43^ Before delving into bifurcations and their representation as normal forms, recall the concept of topological equivalence.

Local topological equivalence between two dynamical systems $\left\{T,R^{n},\varphi^{t}\right\}$ and $\left\{T,R,\psi^{t}\right\}$ is established if there exist a homeomorphism $h:R^{n}\to R^{n}$ that maps orbits of the first system to orbits of the second one, and the direction of time is preserved. Local topologically equivalence near an equilibrium $\hat{u}$ is, in turn, established between a dynamical system $\left\{T,R^{n},\varphi^{t}\right\}$ and a dynamical system $\left\{T,R,\psi^{t}\right\}$ near an equilibrium $\hat{y}$ if there exist a homeomorphism $h:R^{n}\to R^{n}$ that is defined in a small neighborhood $U\in R^{n}$ of $\hat{u}$, satisfies $\hat{y}=h(\hat{u})$, and maps orbits of the $\left\{T,R^{n},\varphi^{t}\right\}\in U$ onto orbits of $\left\{T,R,\psi^{t}\right\}\in V=h\left(U\right)\subset R^{n}$ while preserving the direction of time.

A bifurcation consists in the appearance of a topologically non-equivalent phase portrait under variation of parameters. The difference between the dimension of the parameter space and the dimension of the corresponding bifurcation boundary is called “codimension”.

To determine a system’s behaviour near bifurcations, minimal-order forms, called “normal forms”, can be employed. In fact, the normal form of the bifurcation is locally topologically equivalent near an equilibrium to all systems exhibiting that certain type of bifurcation.^102^

Consider a dynamical system

(Equation 20) $$\dot{x}=f\left(x,p^{\prime }\right),x\in R^{n},p^{\prime }\in R^{n}$$

and a polynomial model

(Equation 21) $$\dot{\zeta }=g\left(\zeta ,p;\beta \right),\zeta \in R^{n},p\in R^{k},\beta \in R^{l}$$

having dimension *n*, codimension *k* and polynomial order *l*. Without loss of generality, a change of coordinates can set the bifurcation point occurs at $\left(x,p\right)=\left(0,p_{0}\right)$.^90^ System (Equation 21) is thus called a *topological normal form* for a given bifurcation if any generic system (Equation 20) with the equilibrium x=0 satisfying the same bifurcation conditions at $p^{\prime }=0$ is locally topologically equivalent near the origin to model (Equation 21) for some values of the coefficients $\beta_{i}$. Using normal forms, it is thus possible to study classes of bifurcations using simple polynomials. If the system satisfies certain conditions on $\partial^{j}f/\partial \varphi^{j}{|}_{\left(0,p_{0}\right)}$ around the critical point, where *j* is the derivative order and φ={x,p}, it is called “generic”. The nondegeneracy conditions $\partial^{j}f/\partial x^{j}$ are related to the “criticality” of a bifurcation,^19^ while the trasversality conditions $\partial^{j}f/\partial p^{j}$ govern the bifurcation unfolding and thus its genericity (the bifurcation exists even after small perturbations). The saddle-node investigated in the main text (*cf.* Figure 4) is the most common generic normal form with dimension 1 and codimension 1.^102^

For low-dimensional systems, their associated normal forms can be explicitly obtained using e.g. Taylor expansion methods over both nondegeneracy and trasversality conditions.^90^ For high-dimensional systems, numerical methods like XPP-AUT (http://www.math.pitt.edu/∼bard/xpp/whatis.html) or network reduction techniques^44^^,^^45^ can be employed to infer or derive the normal forms. Obtaining analytical results for any system is still an open research field.

#### Analysis of slow dynamics

The fluctuations around the stable manifold of Equation 5 can be analysed by studying the fast-slow dynamics around it and determining stochastic equations for the residuals.^19^^,^^49^^,^^68^ Here, we briefly recall the procedure to derive Equation 6. Recall the normal form of a generic fold bifurcation:

(Equation 22) $$\dot{x}=p+x^{2}.$$

It has two steady states:

(Equation 23) $${\hat{x}}_{1}=-\sqrt{-p-0}\;\left(\text{stable}\right)$$

(Equation 24) $${\hat{x}}_{2}=-\sqrt{-p-0}\;\left(\text{unstable}\right)$$

where the term “−0” explicits the distance from the bifurcation point $x_{0}=0$ (by definition). Consider a neighborhood of the attractor (stable fixed point) ${\hat{x}}_{1}$ and see what happens after small perturbations. To do so, perform a local linearization by considering $\delta x=\left(x-{\hat{x}}_{1}\right)$. Thus:

(Equation 25) $$\frac{d\delta x}{dt}≃f\left({\hat{x}}_{1}\right)+\frac{\partial f}{\partial x}{|}_{{\hat{x}}_{1}}\delta x+O\left(\delta x^{2}\right)\text{.}$$

So, using Equation 22 and Equation 23, we obtain:

(Equation 26) $$\frac{d\delta x}{dt}≃2\sqrt{-p}\delta x\;\text{.}$$

This deterministic form con be augmented by a Wiener process with variance σ arbitrary multiplied by h(x), representing non-Gaussian noise properties. This modelling choice converts the family of ODEs into SDEs.^49^^,^^103^^,^^104^ A change δx→y makes the notation lighter into:

(Equation 27) $$dy=2\sqrt{-p}y\;dt+\sqrt{h^{2}\left(x\right)}dW\;\text{.}$$

The equation describes a system evolving under small noise in a neighbourhood of the stable equilibrium, when this is not far away from the bifurcation point.

The term $\left({\hat{x}}_{1}-0\right)=\sqrt{-p}$ is the distance of the stable equilibrium from the bifurcation point and depends on the leading parameter *p*. We can thus rescale it to a new variable −k:

(Equation 28) $$dy=-k\;y\;dt+\sqrt{h^{2}\left(x\right)}dW$$

The sign “−” in “−*k*” is included so that Equation 28 is interpreted as the associated Langevin equation to a Ornstein-Uhlenbeck process.^79^ The term multiplying the deterministic drift can thus be interpreted as −∂V/∂x where V(x) is the potential governing the drift of the particle subjected to random noise. In our case, thanks to the choices made,

(Equation 29) $$V=\frac{1}{2}k\;y^{2}\text{,}$$

that is, a quadratically shaped adjoining potential typical of an overdamped oscillator under noise, of which *k* represents the depth. The working hypothesis is that boundary of the ideal potential *V* can grasp the boundary of the attracting basin of the original model after sufficiently long time. Equation 28 is analytically tractable to understand the main qualitative features of more complicated critical transitions. However, it requires ad hoc extensions when studying system-specific quantitative details like observability boundaries and lead times. Gardiner^79^ also extends Equation 28 to inhomogeneous processes with ramping parameters, used in Equation 13.

#### Reproduce Figure 1

Figure 1 displays examples of a bistable system with critical transitions and hysteresis as well as smooth transitions. Panel (a) corresponds to the bifurcation diagram of Equation 15, flipped along the vertical axis to highlight the hysteresis.

Panel (c) shows the bifurcation diagram, over an unfolded supercritical pitchfork bifurcation, of $\dot{x}=q+p\left(x-1\right)-{\left(x-1\right)}^{3}$, which corresponds to the bifurcation normal form, shifted (to better visualize the diagram) and modified by a small perturbing term q=0.01 unfolding the bifurcation^50^ into a smooth branch. In brief, an *unfolding* of a dynamical system under static equivalence is one that exhibits all possible bifurcations of the equilibrium (rest) points, up to topological equivalence of the set of equilibria.^42^ In other terms, it investigates what happens when small terms are added to the original bifurcation, mimicking extra parameters, small offsets or “impurities”.

The illustrative attractors in panel (a) and (b) are two-well potentials associated, e.g., to the cusp bifurcation (aka “organising center”^50^^,^^93^), a generic bifurcation described by $\dot{x}=a+bx-x^{3}$, where the combination of *a* and *b* determine bistability and the route to a saddle-node bifurcation.

#### Supporting analytical results

##### Entropy in case of Gaussian noise

Within a symmetric potential, elicited by a (locally) quadratic normal form, consider a Gaussian distributed variable y∼N(μ,Var). Its entropy is:

(Equation 30) $$H_{S}\left(y\right)=-\int p\left(y^{\prime }\right)\log\;p\left(y^{\prime }\right)dy^{\prime }==-E\left[\log\;N\left(\mu ,\text{Var}\right)\right]=E\left[\log\left[\frac{1}{\sqrt{2\pi \text{Var}}}\;\exp\;\left(-\frac{1}{2\text{Var}}{\left(x-\mu \right)}^{2}\right)\right]\right]=\frac{1}{2}\log\left(2\pi \text{Var}\right)+\frac{1}{2\text{Var}}E\left[{\left(x-\mu \right)}^{2}\right]==\frac{1}{2}\left[\log\left(2\pi \text{Var}\right)+1\right]\text{,}$$

that is, for the case of Gaussian noise, $H_{S}$ is directly proportional to the variance and displays similar trends, that can be used to derive EWS.

##### Measurement noise

Consider a measurement process with uncertainties $\sigma_{m}^{2}$, independent from system variance (Equation 9). The resulting expected error, obtained from summing the two standard deviation in quadrature,^105^ is:

(Equation 31) $$\sigma_{\text{tot}}^{2}=Var+\sigma_{m}^{2}\;\text{.}$$

To derive the autocorrelation, combine its definition

(Equation 32) $$AC\left(\tau \right)=\frac{Cov\left(x\left(t\right)x\left(t+\tau \right)\right)}{\sqrt{Var\left(x\left(t\right)\right)Var\left(x\left(t+\tau \right)\right)}}=e^{-k\cdot \left|\tau \right|}\;\text{for}\;t\to \infty$$

(where indicates the covariance and Var the variance) with Equation 31 (substituting $Var=\sigma_{\text{tot}}^{2}$). In principle, we can explicitly consider multiplicative noise like in the main text. However, the goal in this case is to compute if notable discrepancies exist between ideal measurements (no uncertainty) and realistic measurements (with some uncertainty, that can be filtered to correspond to additive noise). Hence, only the case of additive white process noise is currently considered. This results in:

(Equation 33) $$AC{\left(1\right)}_{m}=\frac{\frac{\sigma^{2}}{2k}e^{-k}}{\sqrt{{\left(\frac{\sigma^{2}}{2k}+\sigma_{m}^{2}\right)}^{2}}}\text{.}$$

Obviously, $\lim_{\sigma_{m}^{2}\to 0}AC{\left(1\right)}_{m}=AC\left(1\right)$. From Equation 33, we can immediately see that measurement uncertainties $\sigma_{m}$ induce small scaling but do not alter the functional. Only relatively high measurement uncertainty levels change the absolute values of expected lag-1 autocorrelation, but maintain the increasing patterns close to critical points.

#### Skewness and kurtosis

For certain simulated systems, the third statistical moment (skewness) has been suggested to provide useful early warnings.^82^ However, experimental results^26^ were not able to confirm the expectations, estimating flat and fluctuating trends before a tipping point.

For a stochastic process with quasi-steady state parameter and small noise limit, its statistical moments are

(Equation 34) $$\left\langle y^{n}\right\rangle -{\left\langle y\right\rangle }^{n}=\int_{-\infty }^{\infty }{\left(y^{\prime }-\mu \right)}^{n}P\left(y^{\prime }\right)dy^{\prime }$$

where $P\left(y^{\prime }\right)$ is the associated probability density function and μ is the expected average value.

For odd *n*, if μ=0 and $P\left(y^{\prime }\right)$ is symmetric, the integral equals 0 by definition. Symmetric probability density functions are associated, for instance, with quadratic potentials (Equation 29) that are typical of bifurcation normal forms under additive white noise, for which^79^

(Equation 35) $$P\left(y\right)=\sqrt{\frac{k}{\pi \sigma^{2}}}\mathrm{Exp}\left[-\frac{2}{\sigma^{2}}U\left(y\right)\right]=\sqrt{\frac{k}{\pi \sigma^{2}}}\mathrm{Exp}\left[-\frac{ky^{2}}{\sigma^{2}}\right]$$

Consequently, the normal forms considered above, under small noise or in case of symmetric potential, are expected to display a flat skewness.

On the other hand, the integral (.16) may be non-zero, and even dependend on the drift parameter *k*, if μ≠0 or if P(y) is asymmetrical. In the first case, solving Equation 35 yields (provided that Re[k]>0):

(Equation 36) $$\text{Skew}=-\frac{\mu \left(3+2k\mu^{2}\right)}{2k}\;\text{.}$$

In this case, as k→0, the skewness is expected to increase, potentially providing an early warning

On the other hand, an asymmetric potential can be obtained in case of multiplicative noise.^15^^,^^79^ Depending on the specific form, it may be possible to observe increasing trends associated to EWS, but they may be system-specific and not generalisable. In this sense, there is no ambiguity between the results of Guttal et al.^82^ and Dai et al.^26^: they were studying systems with different properties, using an indicator that is not particularly performing and generalisable.

As for the kurtosisn, in case of μ=0 (typical additive white noise), $\text{kurtosis}=3{\text{Var}}^{2}$. This can be obtained by solving Equation 35. If μ≠0, or for other exotic noise forms, and if Re[k]>0:

(Equation 37) $$\text{Kurt}=\frac{3+4k\mu^{2}\left(3+k\mu^{2}\right)}{4k^{2}}\text{,}$$

whose leading term for 0<k<1 still equals ${\mathrm{Var}}^{2}$. Hence, the variance is already representative of higher moments, which are not expected to improve EWS unless system-specific noise and drift forms are considered. Note that, for both (Equation 36), (Equation 37) and (Equation 36), (Equation 37), the constant noise level σ is normalised to 1 for ease of notation.

##### Computational simulations

In all computer simulations of Equation 15, K=0.1 to set bistability. The analysis concentrates on the upper stable branch of the bifurcation diagram (Figure 4, right) to compare with additive white noise results. In this case, multiplicative noise corresponds to intrinsic regulatory mechanisms^76^^,^^106^ rather than stochasticity due to small numbers.^73^ Simulations are performed in Matlab (R2021b) using the Milstein method with a time step of 0.01 (arbitrary units). For quasi-steady state simulations, distribution data for each *c* from far to close the bifurcation point are computed upon stable values of system’s state, after a transient.

The Milstein method runs Monte Carlo chains over Itô-Taylor expanded stochastic differential equations for any variable *z*, up to second order:

(Equation 38) $$z\left(t_{i}+1\right)=z\left(t_{i}\right)+f\left(z\left(t\right)\right)\Delta t+g\left(z\left(t_{i}\right)\right)\Delta W_{i}+\frac{1}{2}g\left(z\left(t_{i}\right)\right)g^{\prime }\left(z\left(t_{i}\right)\right)\left[{\left(\Delta W_{i}\right)}^{2}-\Delta t\right]\text{.}$$

It better converges to the true Itô integral and was proven to have improved accuracy.^107^ When g(z(t))=const (only additive noise without state-dependency), it is equivalent to the common Euler-Maruyama scheme.

Setting simulation parameters of noise intensity and distance to critical points require understanding their reciprocal scales. To do so, we employ a methodology introduced in Kuehn et al.^19^ and Proverbio et al.,^91^ that is, to look for significant changes in the Kramers’ escape rates out of bistable potentials. The Kramers escape rate is^79^

(Equation 39) $$\tau =2\pi {\left(\sqrt{\left|U^{\prime \prime }\left({\tilde{x}}_{1}\right)U^{\prime \prime }\left({\tilde{x}}_{2}\right)\right|}\right)}^{-1}\;\exp\;\left[\left(U\left({\tilde{x}}_{2}\right)-U\left({\tilde{x}}_{1}\right)\right)/\sigma \right]$$

and measures the average expected rate of escape of multiple noisy particles from attracting wells. For any saddle-node bifurcation $\dot{x}=p-x^{2}$ equipped with additive noise, $U\left({\tilde{x}}_{2}\right)-U\left({\tilde{x}}_{1}\right)=32/3p^{3/2}$ and $\left|U^{\prime \prime }\left({\tilde{x}}_{1,2}\right)\right|=2\sqrt{p}$. Hence,

(Equation 40) $$\tau ≃O\left(\exp\;\left[\frac{p^{3/2}}{\sigma }\right]\right)$$

Comparable ranges of control parameters and noise levels are studied in Proverbio et al.^91^ and reproduced in Figure S4. We use those results to distinguish two regimes, one where few noise-induced transitions might occur and another regime primarily determined by the approach to the bifurcation. We set values of $c-c_{0}$ (distance from bifurcation point) and σ (noise intensity) accordingly, to span both regimes and see what changes when n-tipping becomes more frequent.

Finally, the statistical indicators are computed using their standard definitions, using their corresponding MATLAB functions. For example, variance and Shannon entropy $H_{S}$ are:

(Equation 41) $${\text{Var}}_{j}=\frac{1}{N-1}\sum_{r=1}^{N}{\left(B_{j,r}-{\hat{B}}_{j}\right)}^{2}$$

(Equation 42) $$H_{S}=-\sum p_{j}\;\log\;p_{j}$$

for any point *j* corresponding to a single parameter value, with *N* data *B* distributed around a mean value $\hat{B}$ and probability density function $p_{j}$. Other statistical moments and indicators can be computed similarly.

#### p Value assessment of significant increase and optimisation

By theory, an early warning signal is triggered when an increasing trend of suitable statistical indicators is observed.^1^ However, during real-time monitoring, it is often challenging to say whether a measured increase of mean values is significant or not, due to random fluctuations and uncertainties that may occur. If increasing trends are not quantified properly, spurious signals may be triggered.^29^ For analysis performed using moving windows over time-series data, the Kendall’s τ score of monotonous increases have been proposed,^28^^,^^29^ as well as threshold of confidence intervals, with respect to baseline values.^20^

Since we work with distribution data, we propose to employ significance levels on Welch’s p value scores (non-equal variances allowed between the populations), which relate to threshold in confidence intervals and are readily interpreteable.^91^ They also allow to estimate the sensitivity to noise intensities and the expected lead parameter for detection or anticipation of critical transitions. The idea is to compare the full distributions at each parameter value *c* with a reference one, usually taken far from the bifurcation point and without n-tipping, and check whether they are significantly separated toward increasing values. The p value scores are used to assess the significance. This method can still be sensitive to fluctuating scores (hence, a smoothing is employed), but it has the advantage of relying on a-prioristic values, e.g. significant p value $p_{sig}=0.05$. Of course, a p value does not distinguish between increasing or decreasing trends: it is thus coupled with simple visualization of the direction of the trends.

Examples of the three methods are provided in Figure S1.

Quantifying the significance of increasing trends is leveraged as follows: we extract at which value of the control parameter *c* the p value crosses the significance threshold $p_{sig}=0.05$ as a reference. Other common thresholds p=0.1 or p=0.01 can be used, yielding consistent results. When $p-\text{value}<p_{sig}$, it means that an indicator has significantly increased more than the baseline, triggering a warning signal. Consider all $c_{i}$ tested during the simulations, i=1..N with $N=\left(c_{\max}-c_{min}\right)/0.002$; $c_{\max}$ and $c_{\min}$ are two arbitrary values greater and lower than the bifurcation value $c_{0}$, within the bistable region, and 0.002 is the simulation step $\left|c_{i}-c_{i-1}\right|$. Out of all $c_{i}$, estimate $c_{sig}=c_{j}$, where *j* is the first index at which p value <psigj stably, *i.e.*, without considering small fluctuating values (for that, a smoothing is employed). This is performed for each indicator I and each noise level σ. Hence, the analysis estimates

(Equation 43) $$c_{sig}^{I}\left(\sigma \right)=c_{j}s.t.\;p-{\text{value}}_{j}\left(I\right)<p_{sig}\wedge \min\left(j\right)\;\text{.}$$

The optimisation problem described in the main text aims at maximising the combination of all $c_{sig}^{I}\left(\sigma_{l}\right)$ obtained at different noise levels $\sigma_{l}$, so that the results are robust against a range of signal-to-noise ratios. As described in the main text, the analysis is complemented with a counter C to quantify how many tipping events occurred before the bifurcation point, for each σ.

A final comment regards the set of considered indicators I. In principle, CV could be included among the as its performance improves in case of multiplicative noise (see Figure S3. However, the optimisation procedure does not strongly select it, preferring the combinations in Figure 5D. Hence, it has been removed altogether, to improve the computational speed when using more fine-grained steps for the grid search.

### Quantification and statistical analysis

Experimental data were collected and curated by the original study.^26^ We refer to it for details about the experimental protocols. The publicly available data correspond to ensemble of replicate populations, at each observation time corresponding to input dilution factors altering the environmental sucrose concentration. The eight dilution are 250, 500, 750, 1000, 1133, 1266, 1400 and 1600. Population densities were recorded by measuring optical density at 620 nm using a Thermo Scientific Multiskan FC microplate photometer. The values used in the analysis represent cell numbers, estimated from optical densities converted through calibration curves described in thee original publication. For each observation time, several statistical indicators were calculated over the ensembles as explained in the previous section.

The standard errors and confidence intervals of the indicators were given by bootstrap. In bootstrap, the replicates are resampled by combining the data over 5 days (observation lag for one dilution factor) into a single distribution. Resapling was performed by 50 to 1000 repetitions, to check the robustness of final p values against bootstrapping hyper-parameters and to confirm consistency with the original results. Since there are, on average, 60 data entries for each dilution factor value, we eventually employ bootstrapping with 50 repetitions, to avoid biases in the p values due to random over-repetitions of some data.

The p values to quantify significant increases in the distributions of indicators are calculated as described in STAR Methods, using the distribution at dilution factor 250 (the smallest and furthest from the bifurcation point) as baseline, and comparing all other distributions against it, making sure that the mean value was increasing before drawing conclusions.

## Data Availability

• This paper analyzes existing, publicly available data. These accession numbers for the datasets are listed in the key resources table. • All original code has been deposited at Zenodo and is publicly available as of the date of publication. DOIs are listed in the key resources table. • Any additional information required to reanalyze the data reported in this paper is available from the lead contact upon request

## References

[1] Scheffer M., Bascompte J., Brock W.A., Brovkin V., Carpenter S.R., Dakos V., Held H., Van Nes E.H., Rietkerk M., Sugihara G. (2009). Early-warning signals for critical transitions. Nature.

[2] Ashwin P., Zaikin A. (2015). Pattern selection: The importance of ”how you get there”. Biophys. J..

[3] Hirota M., Holmgren M., Van Nes E.H., Scheffer M. (2011). Global Resilience of Tropical Forest. Science.

[4] Wang R., Dearing J.A., Langdon P.G., Zhang E., Yang X., Dakos V., Scheffer M. (2012). Flickering gives early warning signals of a critical transition to a eutrophic lake state. Nature.

[5] Lenton T.M., Livina V.N., Dakos V., Van Nes E.H., Scheffer M. (2012). Early warning of climate tipping points from critical slowing down: comparing methods to improve robustness. Philos. T. R. Soc. A.

[6] Drijfhout S., Bathiany S., Beaulieu C., Brovkin V., Claussen M., Huntingford C., Scheffer M., Sgubin G., Swingedouw D. (2015). Catalogue of abrupt shifts in Intergovernmental Panel on Climate Change climate models. P. Natl. Acad. Sci. USA.

[7] Dmitriev A., Dmitriev V., Sagaydak O., Tsukanova O. (2017). The Application of Stochastic Bifurcation Theory to the Early Detection of Economic Bubbles. Procedia Comput. Sci..

[8] Diks C., Hommes C., Wang J. (2019). Critical slowing down as an early warning signal for financial crises?. Empir. Econ..

[9] Korolev K.S., Xavier J.B., Gore J. (2014). Turning ecology and evolution against cancer. Nat. Rev. Cancer.

[10] Trefois C., Antony P.M.A., Goncalves J., Skupin A., Balling R. (2015). Critical transitions in chronic disease: Transferring concepts from ecology to systems medicine. Curr. Opin. Biotechnol..

[11] Aihara K., Liu R., Koizumi K., Liu X., Chen L. (2022). Dynamical network biomarkers: Theory and applications. Gene.

[12] Quail T., Shrier A., Glass L. (2015). Predicting the onset of period-doubling bifurcations in noisy cardiac systems. P. Natl. Acad. Sci. USA.

[13] Meisel C., Kuehn C. (2012). Scaling effects and spatio-temporal multilevel dynamics in epileptic seizures. PLoS One.

[14] Angeli D., Ferrell J.E., Sontag E.D. (2004). Detection of multistability, bifurcations, and hysteresis in a large class of biological positive-feedback systems. P. Nat. Acad. Sci. USA.

[15] Sharma Y., Dutta P.S., Gupta A.K. (2016). Anticipating regime shifts in gene expression: The case of an autoactivating positive feedback loop. Phys. Rev. E.

[16] Ghaffarizadeh A., Flann N.S., Podgorski G.J. (2014). Multistable switches and their role in cellular differentiation networks. BMC Bioinf..

[17] Mojtahedi M., Skupin A., Zhou J., Castaño I.G., Leong-Quong R.Y.Y., Chang H., Trachana K., Giuliani A., Huang S. (2016). Cell Fate Decision as High-Dimensional Critical State Transition. PLoS Biol..

[18] Lang J., Nie Q., Li C. (2021). Landscape and kinetic path quantify critical transitions in epithelial-mesenchymal transition. Biophys. J..

[19] Kuehn C. (2011). A mathematical framework for critical transitions: Bifurcations, fast–slow systems and stochastic dynamics. Physica D.

[20] Drake J.M., Griffen B.D. (2010). Early warning signals of extinction in deteriorating environments. Nature.

[21] Lade S.J., Gross T. (2012). Early warning signals for critical transitions: a generalized modeling approach. PLoS Comput. Biol..

[22] Chen L., Liu R., Liu Z.P., Li M., Aihara K. (2012). Detecting early-warning signals for sudden deterioration of complex diseases by dynamical network biomarkers. Sci. Rep..

[23] Navid Moghadam N., Nazarimehr F., Jafari S., Sprott J.C. (2020). Studying the performance of critical slowing down indicators in a biological system with a period-doubling route to chaos. Physica A.

[24] Matsumori T., Sakai H., Aihara K. (2019). Early-warning signals using dynamical network markers selected by covariance. Phys. Rev. E.

[25] Carpenter S.R., Cole J.J., Pace M.L., Batt R., Brock W.A., Cline T., Coloso J., Hodgson J.R., Kitchell J.F., Seekell D.A. (2011). Early warnings of regime shifts: A whole-ecosystem experiment. Science.

[26] Dai L., Vorselen D., Korolev K.S., Gore J. (2012). Generic indicators for loss of resilience before a tipping point leading to population collapse. Science.

[27] Wilkat T., Rings T., Lehnertz K. (2019). No evidence for critical slowing down prior to human epileptic seizures. Chaos.

[28] Proverbio D., Kemp F., Magni S., Gonçalves J. (2022). Performance of early warning signals for disease re-emergence: A case study on COVID-19 data. PLoS Comput. Biol..

[29] Boettiger C., Hastings A. (2012). Quantifying limits to detection of early warning for critical transitions. J. R. Soc. Interface.

[30] Clements C.F., Ozgul A. (2018). Indicators of transitions in biological systems. Ecol. Lett..

[31] Dudney J., Suding K.N. (2020). The elusive search for tipping points. Nat. Ecol. Evol..

[32] Kuehn C., Lux K., Neamtu A. (2022). Warning Signs for Non-Markovian Bifurcations: Color Blindness and Scaling Laws. P. Roy. Soc. A.

[33] Cohen A.A., Leung D.L., Legault V., Gravel D., Blanchet F.G., Côté A.M., Fülöp T., Lee J., Dufour F., Liu M., Nakazato Y. (2022). Synchrony of biomarker variability indicates a critical transition: Application to mortality prediction in hemodialysis. iScience.

[34] Mazzocchi F. (2012). Complexity and the reductionism–holism debate in systems biology. Wires Syst. Biol. Med..

[35] Stumpf P.S., Smith R.C.G., Lenz M., Schuppert A., Müller F.J., Babtie A., Chan T.E., Stumpf M.P.H., Please C.P., Howison S.D. (2017). Stem cell differentiation as a non-markov stochastic process. Cell Syst..

[36] Ferrell J.E., Pomerening J.R., Kim S.Y., Trunnell N.B., Xiong W., Huang C.-Y.F., Machleder E.M. (2009). Simple, realistic models of complex biological processes: positive feedback and bistability in a cell fate switch and a cell cycle oscillator. FEBS Lett..

[37] Moris N., Pina C., Arias A.M. (2016). Transition states and cell fate decisions in epigenetic landscapes. Nat. Rev. Genet..

[38] Maini P.K., Myerscough M.R., Winters K.H., Murray J.D. (1991). Bifurcating spatially heterogeneous solutions in a chemotaxis model for biological pattern generation. Bull. Math. Biol..

[39] Yasemi M., Jolicoeur M. (2021). Modelling cell metabolism: a review on constraint-based steady-state and kinetic approaches. Processes.

[40] Del Vecchio D., Dy A.J., Qian Y. (2016). Control theory meets synthetic biology. J. R. Soc. Interface.

[41] MacArthur B.D., Ma’ayan A., Lemischka I.R. (2009). Systems biology of stem cell fate and cellular reprogramming. Nat. Rev. Mol. Cell Biol..

[42] Kuznetsov Y.A. (2013).

[43] Kuehn C., Bick C. (2021). A universal route to explosive phenomena. Sci. Adv..

[44] Gao J., Barzel B., Barabási A.L. (2016). Universal resilience patterns in complex networks. Nature.

[45] Tu C., D’Odorico P., Suweis S. (2021). Dimensionality reduction of complex dynamical systems. iScience.

[46] Tsimring L.S. (2014). Noise in biology. Rep. Prog. Phys..

[47] Su Y., Bintz M., Yang Y., Robert L., Ng A.H.C., Liu V., Ribas A., Heath J.R., Wei W. (2019). Phenotypic heterogeneity and evolution of melanoma cells associated with targeted therapy resistance. PLoS Comput. Biol..

[48] Zhang H., Chen Y., Chen Y. (2012). Noise Propagation in Gene Regulation Networks Involving Interlinked Positive and Negative Feedback Loops. PLoS One.

[49] Berglund N., Gentz B. (2006). Noise-induced Phenomena in Slow-Fast Dynamical Systems: A Sample-Paths Approach.

[50] Thompson J.M.T., Sieber J. (2011). Predicting climate tipping as a noisy bifurcation: a review. Int. J. Bifurcation Chaos.

[51] Ashwin P., Wieczorek S., Vitolo R., Cox P. (2012). Tipping points in open systems: bifurcation, noise-induced and rate-dependent examples in the climate system. Phil. Trans. Roy. Soc. A.

[52] Shi J., Li T., Chen L. (2016). Towards a critical transition theory under different temporal scales and noise strengths. Phys. Rev. E.

[53] Ozbudak E.M., Thattai M., Lim H.N., Shraiman B.I., Van Oudenaarden A. (2004). Multistability in the lactose utilization network of Escherichia coli. Nature.

[54] Dai L., Korolev K.S., Gore J., Carpenter S.R. (2015). Relation between stability and resilience determines the performance of early warning signals under different environmental drivers. P. Natl. Acad. Sci. USA.

[55] Sarkar S., Sinha S.K., Levine H., Jolly M.K., Dutta P.S. (2019). Anticipating critical transitions in epithelial-hybrid-mesenchymal cell-fate determination. P. Natl. Acad. Sci. USA.

[56] Izhikevich E.M. (2007).

[57] Sornette D. (2006).

[58] Antoniou D., Schwartz S.D. (2011). Protein dynamics and enzymatic chemical barrier passage. J. Phys. Chem. B.

[59] Horsthemke W., Lefever R. (1984). Noise-Induced Transitions in Physics, Chemistry, and Biology.

[60] Wieczorek S., Ashwin P., Luke C.M., Cox P.M. (2011). Excitability in ramped systems: The compost-bomb instability. Proc. R. Soc. A.

[61] Bonciolini G., Ebi D., Boujo E., Noiray N. (2018). Experiments and modelling of rate-dependent transition delay in a stochastic subcritical bifurcation. R. Soc. Open Sci..

[62] Moejes F.W., Matuszyńska A., Adhikari K., Bassi R., Cariti F., Cogne G., Dikaios I., Falciatore A., Finazzi G., Flori S. (2017). A systems-wide understanding of photosynthetic acclimation in algae and higher plants. J. Exp. Bot..

[63] Zhou J.X., Aliyu M.D.S., Aurell E., Huang S. (2012). Quasi-potential landscape in complex multi-stable systems. J. R. Soc. Interface.

[64] Andrecut M., Halley J.D., Winkler D.A., Huang S. (2011). A general model for binary cell fate decision gene circuits with degeneracy: Indeterminacy and switch behavior in the absence of cooperativity. PLoS One.

[65] Stanoev A., Schröter C., Koseska A. (2021). Robustness and timing of cellular differentiation through population-based symmetry breaking. Development.

[66] Wang J., Zhang K., Xu L., Wang E. (2011). Quantifying the Waddington landscape and biological paths for development and differentiation. P. Nat. Acad. Sci. USA.

[67] Alon U. (2006).

[68] O’Regan S.M., Burton D.L. (2018). How stochasticity influences leading indicators of critical transitions. Bull. Math. Biol..

[69] Liu X.M., Xie H.Z., Liu L.G., Li Z.B. (2009). Effect of multiplicative and additive noise on genetic transcriptional regulatory mechanism. Physica A.

[70] Sidney R.C., Dunlop M., Elowitz M.B. (2010). A synthetic three-color reporter framework for monitoring genetic regulation and noise. J. Biol. Eng..

[71] Wang X., Li L., Cheng Y., Liu Q. (2012). Construction of gene regulatory networks with colored noise. Neural Comput. Appl..

[72] Boettiger C. (2018). From noise to knowledge: how randomness generates novel phenomena and reveals information. Ecol. Lett..

[73] Gillespie D.T. (2000). Chemical Langevin equation. J. Chem. Phys..

[74] Allen L.J.S. (2010).

[75] Van Kampen N.G. (1992).

[76] Hasty J., Pradines J., Dolnik M., Collins J.J. (2000). Noise-based switches and amplifiers forgene expression. P. Natl. Acad. Sci. USA.

[77] Holling C.S. (1996). Engineering resilience versus ecological resilience. Engineering within ecological constraints.

[78] Kitano H. (2004). Biological robustness. Nat. Rev. Genet..

[79] Gardiner C.W. (1985).

[80] Dunlop M.J., Cox R.S., Levine J.H., Murray R.M., Elowitz M.B. (2008). Regulatory activity revealed by dynamic correlations in gene expression noise. Nat. Genet..

[81] Bury T.M., Bauch C.T., Anand M. (2020). Detecting and distinguishing tipping points using spectral early warning signals. J. R. Soc. Interface.

[82] Guttal V., Jayaprakash C. (2008). Changing skewness: An early warning signal of regime shifts in ecosystems. Ecol. Lett..

[83] Kéfi S., Dakos V., Scheffer M., Van Nes E.H., Rietkerk M. (2013). Early warning signals also precede non-catastrophic transitions. Oikos.

[84] Boettiger C., Ross N., Hastings A. (2013). Early warning signals: The charted and uncharted territories. Theor. Ecol..

[85] Dakos V., Carpenter S.R., van Nes E.H., Scheffer M. (2015). Resilience indicators: Prospects and limitations for early warnings of regime shifts. Phil. Trans. R. Soc. B..

[86] Pavithran I., Sujith R.I. (2021). Effect of rate of change of parameter on early warning signals for critical transitions. Chaos.

[87] Brett T.S., Drake J.M., Rohani P. (2017). Anticipating the emergence of infectious diseases. J. R. Soc. Interface.

[88] Feng S., Sáez M., Wiuf C., Feliu E., Soyer O.S. (2016). Core signalling motif displaying multistability through multi-state enzymes. J. R. Soc. Interface.

[89] Weber M., Buceta J. (2013). Stochastic stabilization of phenotypic states: the genetic bistable switch as a case study. PLoS One.

[90] Strogatz S.H. (2015).

[91] Proverbio D., Montanari A.N., Skupin A., Gonçalves J. (2022). Buffering variability in cell regulation motifs close to criticality. Phys. Rev. E.

[92] Rosso O.A., Larrondo H.A., Martin M.T., Plastino A., Fuentes M.A. (2007). Distinguishing noise from chaos. Phys. Rev. Lett..

[93] Marco E., Karp R.L., Guo G., Robson P., Hart A.H., Trippa L., Yuan G.C. (2014). Bifurcation analysis of single-cell gene expression data reveals epigenetic landscape. P. Natl. Acad. Sci. USA.

[94] Clements C.F., Ozgul A. (2016). Including trait-based early warning signals helps predict population collapse. Nat. Commun..

[95] Bury T.M., Sujith R.I., Pavithran I., Scheffer M., Lenton T.M., Anand M., Bauch C.T. (2021). Deep learning for early warning signals of tipping points. P. Natl. Acad. Sci. USA.

[96] Laurence E., Doyon N., Dubé L.J., Desrosiers P. (2019). Spectral Dimension Reduction of Complex Dynamical Networks. Phys. Rev. X.

[97] Heino M.T.J., Proverbio D., Marchand G., Resnicow K., Hankonen N. (2022). Attractor landscapes: A unifying conceptual model for understanding behaviour change across scales of observation. Health Psychol. Rev..

[98] Weinans E., Quax R., van Nes E.H., Leemput I.A.v.d. (2021). Evaluating the performance of multivariate indicators of resilience loss. Sci. Rep..

[99] Dakos V., Scheffer M., van Nes E.H., Brovkin V., Petoukhov V., Held H. (2008). Slowing down as an early warning signal for abrupt climate change. P. Nat. Acad. Sci. USA.

[100] Chen N., Jayaprakash C., Yu K., Guttal V. (2018). Rising variability, not slowing down, as a leading indicator of a stochastically driven abrupt transition in a dryland ecosystem. Am. Nat..

[101] Deb S., Bhandary S., Sinha S.K., Jolly M.K., Dutta P.S. (2022). Identifying critical transitions in complex diseases. J. Biosci..

[102] Haragus M., Iooss G. (2010).

[103] Namachchivaya N.S., Leng G. (1990). Equivalence of stochastic averaging and stochastic normal forms. J. Appl. Mech..

[104] Khas’minskii R.Z. (1966). A limit theorem for the solutions of differential equations with random right-hand sides. Theory Probab. Appl..

[105] Taylor J.R. (1997).

[106] Norman T.M., Lord N.D., Paulsson J., Losick R. (2015). Stochastic switching of cell fate in microbes. Annu. Rev. Microbiol..

[107] Bayram M., Partal T., Buyukoz G.O. (2018). Numerical methods for simulation of stochastic differential equations. Adv. Differ. Equ-NY.

